# Optical Coherence Tomography Angiography in Healthy Adult Subjects: Normative Values, Frequency, and Impact of Artifacts

**DOI:** 10.1155/2022/7286252

**Published:** 2022-03-07

**Authors:** Nakhleh E. Abu-Yaghi, Abdelrahman F. Obiedat, Tamara I. AlNawaiseh, Ali M. Hamad, Basil A. Bani Ata, Ahmad A. Quzli, Saif Aldeen AlRyalat

**Affiliations:** ^1^Department of Special Surgery, Ophthalmology Division, School of Medicine, The University of Jordan, Amman, Jordan; ^2^School of Medicine, The University of Jordan, Amman, Jordan

## Abstract

**Aim:**

This cross-sectional study is aimed at identifying normative ocular coherence tomography angiography (OCTA) values in a cohort of healthy adult Jordanian individuals and assessing the prevalence of different image artifacts and their impact on quantitative OCTA measurements.

**Materials and Methods:**

One hundred and eighty-one eyes from 100 healthy participants were included in this study. All participants underwent a comprehensive ophthalmological examination including best corrected visual acuity, slit lamp examination, and dilated fundoscopy. Swept-source OCTA images were obtained and analyzed for all 181 eyes. We recorded vascularity measurements and analyzed the prevalence and effect of ten different artifacts on superficial and deep retinal and choriocapillaris layer images.

**Results:**

Sixty-two percent of the participants were men (*n* = 62), and 38% (*n* = 38) were women. The age of participants ranged between 24 and 75 years (mean 50.5 ± 10.92). The mean central macular thickness was 237.71 (±22.905) *μ*m, and the mean choroidal thickness was 257.73 (±77.027) *μ*m. Artifacts were present in 46.4% of the acquired scans. Images with artifacts had higher mean age (*p* = 0.03), lower image quality (*p* < 0.001), higher central vascular density (*p* < 0.001), and lower inferior vascular density (*p* < 0.001) compared to artifact-free tomographs. Motion artifact was the most common type, which was present in 29 (16%) of images, followed by blink artifact 18 (9.9%), and Z offset 8 (4.4%).

**Conclusion:**

OCTA artifact detection and correction remains a challenging aspect of the diagnostic and follow-up process of patients with retinal pathologies. To our knowledge, this is the first study to examine the association between OCTA outputs and artifacts in healthy eyes. We report that in this cohort of normal individuals, images with artifacts had a significantly higher central vascular density (22.62 vs. 16.60) and a lower inferior vascular density (46.09 vs. 48.81). We also found that a significant increase in central vascular density is only present in images with Z offset artifact type (49.03). Motion artifact was the most common artifact seen in our series. However, we observed no alteration in quantitative parameters in images with motion artifacts.

## 1. Introduction

Optical coherence tomography angiography (OCTA) is an imaging modality for visualization of ocular vessels by detecting motion contrast from flowing blood [1]. Compared to previous dye-based imaging tests, such as fluorescein angiography, OCTA is noninvasive and time-efficient and provides for three-dimensional examination of retinal vasculature [2]. This allows for visualization of the various retinal blood layers including the deep retinal plexus providing both quantitative and qualitative measurements [3, 4]. There are many ways to visualize flow in the retinal vessels, and like any other imaging modality, additional undesirable artifacts can surface on the acquired scan. These artifacts can affect quantitative and qualitative outputs, making image interpretation more challenging. Common, well-described artifacts have been reported, including defocus, shadow, motion, segmentation, tilt, and projection [5–7]. It is proposed that artifact severity and frequency vary depending on different retinal pathologies [8, 9]. The most commonly described type of artifact is the motion artifacts. These may occur both in the axial and transverse tomographs depending on eye movement direction [10]. Although improvements in OCTA algorithms have significantly reduced the effect of aberrations like projection and motion, shadow and defocus remain amongst the most troublesome artifacts that influence the quantitative outputs [11–13]. Artifacts will also affect qualitative image analysis as they diminish recognition of nonperfusion areas outside the macula, with wide-field images inviting more aberrations that make peripheral scans even more difficult to interpret. Identifying artifacts correctly and understanding their frequency and correlation to different pathologies will guide future development of new algorithms that eliminate such confounders and yield more accurate and error-free images. Failure to properly recognize those erroneous images may lead to incorrect diagnosis and management of retinal diseases, and image artifacts may lead to false-positive diagnoses in healthy eyes. Normative OCTA values for Middle Eastern populations are scarce, and image artifacts in normal eyes have not been well studied. Therefore, establishing an accurate analysis of such images is essential as OCTA is being more widely used in clinical settings as well as clinical trials. This cross-sectional study is aimed at filling the current gap by identifying OCTA parameters in a normal healthy Middle Eastern population and assessing the prevalence of different artifacts found in healthy eyes and their influence on quantitative OCTA measurements.

## 2. Materials and Methods

### 2.1. Study Settings and Participants

This cross-sectional study was conducted at Jordan University Hospital between November 1, 2020, and November 1, 2021. The study included 100 consecutive adult participants and a total of 181 healthy eyes. Patients presenting to the eye clinic underwent a complete ophthalmic examination and were invited to participate in the study by signing a written consent form. Sixty-two of the participants were men, and 38 were women. The age of participants ranged between 24 and 75 years (mean 50.5 ± 10.92). Participants with macular or retinal pathologies, refractive errors of more than three diopters, abnormalities on OCTA, or previous history of vitreoretinal surgery were excluded from the study. Eyes with missing parameters and eyes that could not be imaged (e.g., ocular media opacity) were also excluded from the study.

### 2.2. Ethical Considerations

All participants were informed about the nature of the study, and a written consent was obtained prior to enrollment. The research protocol was approved by the Institutional Review Board (IRB) at Jordan University Hospital. All procedures contributing to this work comply with the tenets of the Helsinki Declaration.

### 2.3. Imaging Protocol and Assessments

All participants underwent a comprehensive ophthalmological examination including best corrected visual acuity, slit lamp examination, and dilated fundoscopy. Thereafter, OCTA examination was performed by an expert technician, and OCTA parameters were recorded and analyzed for all 181 eyes. We assessed the presence and type of artifacts on OCTA images, including superficial, deep, outer retinal, and choriocapillaris images. Artifact assessment was performed by an experienced ophthalmologist, whereas the quality index and vascularity measurements were extracted by other authors to ensure accurate assessment of artifact presence and type.

All participants underwent OCTA imaging using a swept-source ocular coherence tomography (OCT) machine (DRI OCT Triton; Topcon Inc, Tokyo, Japan) with a wavelength of 1050 nm and an acquisition speed of 100,000 A-scans per second. This provides a lateral resolution of 20 *μ*m and an in-depth resolution of 2.6 *μ*m. A series of quantitative OCT and angiographic metrics of macular thickness, choroidal thickness, and vascularity indices were measured by the program automatically. Individual OCTA images were generated by IMAGEnet6 (v.1.27.17368, License: 1), which was also used to assess image equality. Scan scales were 4.5 × 4.5 mm.

**Figure 1:**
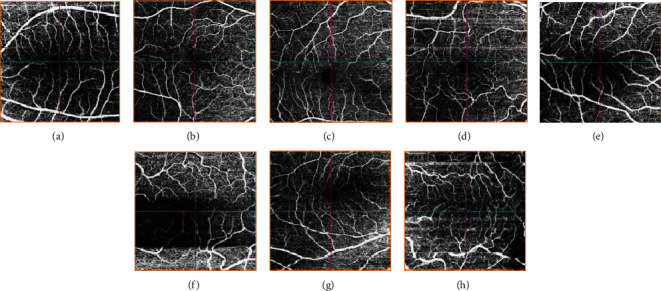
Ocular coherence tomography angiography sample artifacts: (a): defocus; (b): tilt; (c): Z offset; (d): motion; (e): shadow; (f): blink; (g): decentration; (h): segmentation.

Adopting from the Holmen et al. study [14], we tracked the following artifacts (Figure 1):

1. *Decentration artifact* occurs when the scan is not centered on the macula so that the central or inner subfields were outside the OCT grid after repositioning by at least 10%
2. *Segmentation error artifact* is defined as an error in detecting the correct position of retinal boundaries and occurs when the algorithm-generated segmentation line is classified as an error if the line deviates by more than 50% of the thickness of the pertinent plexus. We assessed segmentation errors by observing images generated for superficial, deep, outer retinal, and choriocapillaris angiography images
3. *Eye movement artifact* is defined as one or more of the following: thin vertical or horizontal white lines over the angiogram in conjunction with interruption, displacement, doubling or ghosting of vessels, and/or quilting defect. Eye movement can result in missing areas of the retina as well as duplicated areas of the retina in the scan
4. *Defocus artifact* refers to the decrease in reflective intensity of the entire B scan and global loss of small capillary vessels on angiogram
5. *Blink artifact* is defined by the presence of horizontal black band indicating missing scans
6. *Refractive shift artifact* is a change in reflective intensity between contiguous OCTA scans owing to blinking and a change in refractive index on the corneal surface
7. *Shadow artifact* has decreased intensity of retinal layers in isolated areas, often owing to vitreous floaters or corneal opacities
8. *Z offset artifact* is characterized by a cross-sectional OCT scan vertically displaced in the OCT window owing to a faulty head placement (also termed “out of window”)
9. *Tilt artifact* is identified by only one-half of the cross-sectional OCT scan being in focus; this artifact occurs because of a severe angle of incidence, head placement, and/or high myopia
10. *The projection artifact* is identified by assessing the deep, avascular, and choriocapillaris layers for artifactual vessels projected from superficial layers

### 2.4. Statistical Analysis

We used SPSS version 26.0 (Chicago, USA) in our analysis. We used mean (± standard deviation) to describe continuous variables. We used count (frequency) to describe other nominal variables. We performed independent sample *t*-test to analyze the mean difference between presence and absence of artifact and each continuous measurement (e.g., quality index, vascularity, and thickness measurements), and we also performed one-way ANOVA with Tukey post hoc analysis for each type of artifact and continuous measurements. We performed generalized estimation equation (GEE) to analyze the relation between the presence of artifacts and each continuous measurement on OCT (e.g., quality index, vascularity, and thickness measurements), where we accounted for interrelated data during the analysis. We presented data in mean difference and standard deviation (SD). All underlying assumptions were met. We adopted a *p* value of 0.05 as a significant threshold.

## 3. Results

A total of 100 patients were included in this study, with a mean age of 50.5 (±10.92) years. They were 62 (62%) men and 38 (38%) women. OCTA imaging data were available for 181 eyes.  Table 1 describes the characteristics of the studied sample.

Normal values of macular thickness, choroidal thickness, and vascularity indices are shown in Table 2.

As we compared the characteristics of images with and without artifacts, we found that images with artifacts had higher mean age (*p* = 0.03), lower image quality (*p* < 0.001), higher central vascular density (*p* < 0.001), and lower inferior vascular density (*p* < 0.001), as shown in Table 3.

Upon performing generalized estimation equation (GEE) to compare different measurements with the presence and absence of artifact, accounting for multiple measurements for the same subject (i.e., both eyes), we found significant difference between presence of artifact and central vascular density (*B* = 6.02, 95% confidence interval 2.74 to 9.31, and *p* < 0.001), inferior vascular density (*B* = 2.72, 95% confidence interval 1.12 to 4.32, and *p* = 0.001), and image quality (*B* = 5.59, 95% confidence interval 3.56 to 7.62, and *p* < 0.001). We found no significant relation with central macular thickness (*p* = 0.65), choroidal thickness (*p* = 0.90), superior vascular density (*p* = 0.35), nasal vascular density (*p* = 0.162), or temporal vascular density (*p* = 0.19).

**Figure 2:**
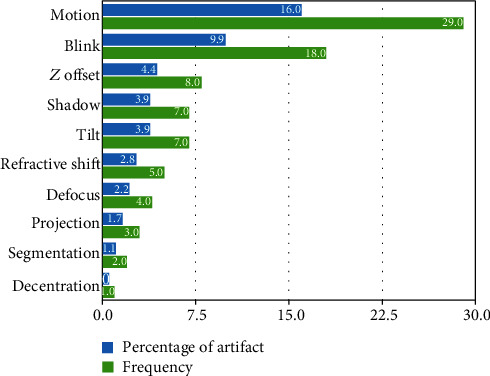
The overall frequency and percentage of each artifact type.

Motion artifact was the most common type, present in 29 (16%) of images, followed by blink artifact 18 (9.9%), and Z offset 8 (4.4%).  Figure 2 shows the overall percentage of each artifact type.

Image quality as assessed by the OCT machine significantly differed between images with and without artifact, as it was 64.4 (SD 4.28) for images without artifact, compared to only 58.8 (SD 7.98) for images with artifact (*p* < 0.001). On post hoc analysis, we found that image quality with blink artifact (56.1 ± 7.79) and motion artifact (59.9 ± 8.06) was the only one significantly lower versus images without artifacts (*p* values 0.001 and 0.018, respectively).

**Figure 3:**
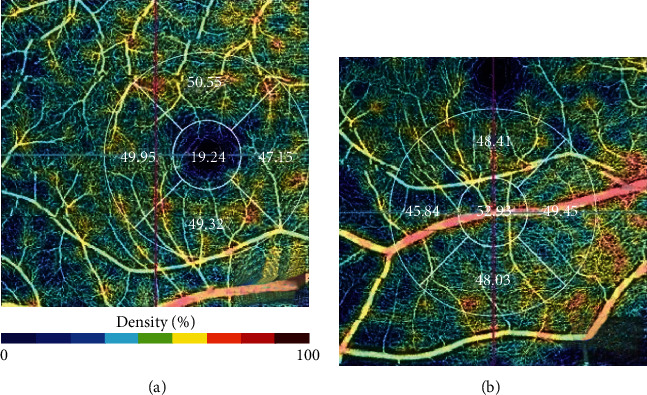
(a) Central vascularity index should be placed in the foveal avascular zone. (b) A shift to other areas will lead to higher measurements, leading to Z offset artifact.

Upon comparing the central vascularity index between each type of artifact and images without artifact, we found a significant difference only for Z offset artifact type (*p* < 0.001), where central vascularity index for images without artifact was 16.6 (SD 4.36), compared to 49.03 (SD 4.87) for images with Z offset artifact.  Figure 3 shows how central vascularity index should be measured in the foveal avascular zone (a), and how placing it in other areas will lead to higher measurements (b), leading to Z offset artifact.

## 4. Discussion

OCTA is an emerging modality used to detect and diagnose vascular pathologies and abnormalities in the posterior segment of the eye. However, despite its promising potential, there remains to be scarcity in baseline data and normal values of retinal and choroidal vascular parameters. Our research provides normal values of vascular densities, as well as macular and choroidal thickness in 181 healthy eyes in a Middle Eastern population.

In our study, the mean central macular thickness was 237.71 (±22.905) *μ*m, and these values are similar to the ones previously reported in the literature in Middle Eastern subjects using the Fournier domain OCT, 229.5 (±30.85) *μ*m [15]. The mean choroidal thickness was found to be 257.73 (±77.027) *μ*m in our cohort. In their studies on healthy eyes, Manjunath et al. and Margolis and Spaide reported a mean choroidal thickness of 272 (±81.0) *μ*m and 287 (±76.0) *μ*m, respectively [16, 17]. These minor variations in choroidal thickness can be attributed to suboptimal number of averaged OCT-B scans and the lack of eye tracking software in older OCT models.

Although vascular densities have been well described in diseased eyes [18–21], there is a lack of normal values of vascular densities in healthy eyes. Such data can be useful in the early detection of retinal abnormalities. To our knowledge, this is the first study that establishes normal values of vascular densities in a Middle Eastern Arab population.

OCTA is a noninvasive technique that can provide images of the retinal and choroidal vascular trees. Like other retinal imaging modalities, OCTA artifacts are common and originate in relation to image acquisition, processing algorithms, certain characteristics of the eye, certain retinal pathologies, and eye movement during image capture. In our study, artifacts were present in 46.4% of images. There has been substantial variation in the prevalence of artifacts in the literature; this is mainly due to two important factors: the machine model and the presence of underlying retinal diseases in participants. These factors have been well studied in the literature [6, 22–25].

The study by Holmen et al. showed that artifacts were present in 97% of OCTA images [14]. Han and Jaffe reported artifacts in 84.7% of Cirrus volume scans and 90.9% of Spectralis scans [25]. Enders et al. observed a similar percentage, where 100% of the 75 imaged eyes showed artifacts [26]. Falavarjani et al., who also used a swept-source OCT, observed artifacts in 89.4% of imaged eyes [27]. This can be explained by the nature of the study population. Whereas we included 181 healthy eyes, Falavarjani only included 12 healthy eyes and 45 eyes with retinal pathologies.

In our study, motion artifact was the most prevalent artifact, which was present in 16% of images, followed by blink artifact 9.9%, and Z offset 4.4%. This is slightly different than what has been reported. Holmen et al. found that motion, defocus, and shadow artifact were the most prevalent in untreated eyes with diabetic retinopathy [14]. However, Ender's study, which included healthy and diseased eyes, revealed projection artifacts to be the most common, followed by segmentation and motion [26]. Falavarjani et al. found that the most prevalent types of artifacts were banding, segmentation, and motion in a cohort involving healthy eyes and eyes with diabetic retinopathy, age-related macular degeneration, and venous occlusive disease [27]. The reason behind such variations is that the type of artifact is dependent on many factors including image acquisition, intrinsic ocular characteristics/pathologies, eye motion, image processing, and display strategies [5, 27].

Limited research exists on the association between artifacts and quantitative outputs of OCTA. To our knowledge, this is the first study where association between OCTA outputs and artifacts is being studied in healthy eyes. Upon comparing the characteristics of images with and without artifacts, we found that images with artifacts had a significantly higher central vascular density (22.62 vs. 16.60) and a lower inferior vascular density (46.09 vs. 48.81). We also found that a significant increase in central vascular density is only present in images with Z offset artifact type (49.03). Our most common artifact was motion; such artifact leads to reporting missing and duplicated areas of the retina in the scan. However, we observed no alteration in quantitative parameters in images with motion artifacts.

OCTA artifact detection and correction remains a challenging aspect of the diagnostic and follow-up process of patients with retinal pathologies. However, prevention of such artifacts seems theoretically feasible, mainly through continuous hardware maintenance, patient coaching, and technician training. We have found that motion, blink, and Z offset artifacts occurred most frequently. Recently, motion correction technology has been developed to prevent motion artifacts. Like other OCT models, Topcon has implemented a retinal eye tracking system (SMARTTRACK) that actively follows eye movements, which in turn decreases the occurrence of motion artifacts and eliminates the need of motion correction software [11]. On the other hand, blink artifacts can be easily averted through effective patient teaching and communication throughout the imaging process. Z offset artifact occurs when OCT scans are vertically displaced in the OCT window owing to a faulty head placement. It was the third most observed artifact in our study. It is of certain importance because we found that the quality index will not be affected when there is a Z offset artifact, making it harder to be detected. To prevent such artifact, a warning should appear when the central indicator, normally lying within the foveal avascular zone area, detects higher vascularity or is moved closer to peripheral areas.

It is worth mentioning that swept-source technology has developed many promising features that can potentially eliminate artifacts. For example, it provides the world's fastest 100,000 A-scans per second, thus reducing involuntary eye movement error. Moreover, during measurement, a 1050 nm wavelength light helps to reduce involuntary eye movement. This longer wavelength is less susceptible to light scattering which decreases the frequency of projection artifacts. This, along with the healthy recruited participants, can partially explain the relatively lower prevalence of artifacts in our report. Our study did not address the effect of mydriasis on different artifacts. In general, motion artifact improves with dilatation of the pupil. Nevertheless, OCTA image metrics taken with different pupillary states are valid for clinical trials [28]. Future studies can overcome the limitations of this report by including larger number of patients from different ethnicities to provide more representative normal data and by comparing the frequency of artifacts between different scan scales. An even further step would look into the repeatability of artifacts in the same subjects whenever the scans are reproduced multiple times.

## 5. Conclusion

OCTA is a relatively new modality used to image patients with retinal diseases. Data on normal values and the impact of artifacts are still evolving. This study included OCTA images for adult patients without retinal disease and established means and ranges for OCTA-based indices such as central macular thicknesses and retinal and choroidal vascular densities for artifact-free images. The frequency of ten different OCTA artifacts along with their impact on image quality and measured indices was reported and analyzed. The most common artifact was motion artifact. Image quality was only affected in blink and motion artifacts, while central vascularity index was affected only in Z offset artifact type.

## Figures and Tables

**Table 1:** Characteristics of the study sample including normative OCTA values and the types of artifacts.

	Count	Column *N* %	Mean	Standard deviation
Laterality				
Left	91	50.3%		
Right	90	49.7%		
Image quality			62	7
Central vascular density			19.28	9.19
Presence of artifact				
No	97	53.6%		
Yes	84	46.4%		
Decentration	1	0.6%		
Segmentation	2	1.1%		
Motion	29	16.0%		
Defocus	4	2.2%		
Blink	18	9.9%		
Refractive shift	5	2.8%		
Shadow	7	3.9%		
Z offset	8	4.4%		
Tilt	7	3.9%		
Projection	3	1.7%		
Central macular thickness (*μ*m)			237	25
Choroidal thickness (*μ*m)			257	74
Superior vascular density			48.70	5.76
Nasal vascular density			45.99	4.74
Inferior vascular density			47.58	5.12
Temporal vascular density			45.29	5.57

**Table 2:** Mean, standard deviation, minimum, and maximum values of anatomical and vascular characteristics of normal images without any artifacts.

	Minimum	Maximum	Mean	Std. deviation
Central macular thickness (*μ*m)	182	292	237.71	22.905
Choroidal thickness (*μ*m)	29	453	257.73	77.027
Central vascular density	8.10	29.00	16.5995	4.36490
Superior vascular density	39.02	56.30	49.1363	3.53108
Nasal vascular density	33.62	57.80	46.5011	4.62511
Inferior vascular density	39.52	60.73	48.8080	4.33395
Temporal vascular density	35.50	58.70	45.8717	5.16849

**Table 3:** Comparison of image characteristics with and without artifacts.

	Presence of artifact						*p* value
	No			Yes			
	Count	Mean	Standard deviation	Count	Mean	Standard deviation	
Laterality							
Left	49 (50.5%)			42 (50%)			
Right	48 (49.5%)			42 (50%)			
Age		49.06	9.30		52.48	11.79	0.03
Gender							
Male	61 (62.9)			53 (63.1%)			
Female	36 (37.1%)			31 (36.9%)			
Image quality		64.41	4.28		58.82	7.98	<0.001
Central vascular density		16.60	4.36		22.62	12.13	<0.001
Central macular thickness (*μ*m)		237.71	22.91		235.81	27.47	0.62
Choroidal thickness (*μ*m)		257.73	77.03		256.11	70.53	0.89
Superior vascular density		49.14	3.53		48.17	7.63	0.30
Nasal vascular density		46.50	4.63		45.38	4.84	0.12
Inferior vascular density		48.81	4.33		46.09	5.60	<0.001
Temporal vascular density		45.87	5.17		44.58	5.98	0.13

## Data Availability

The datasets generated and/or analyzed during the current study are available from the corresponding author upon reasonable request.
